# Psychology Meets Archaeology: Psychoarchaeoacoustics for Understanding Ancient Minds and Their Relationship to the Sacred

**DOI:** 10.3389/fpsyg.2020.550794

**Published:** 2020-12-18

**Authors:** Jose Valenzuela, Margarita Díaz-Andreu, Carles Escera

**Affiliations:** ^1^Brainlab ‐ Cognitive Neuroscience Research Group, Department of Clinical Psychology and Psychobiology, Faculty of Psychology, University of Barcelona, Barcelona, Spain; ^2^Institute of Neurosciences, University of Barcelona, Barcelona, Spain; ^3^Catalan Institution for Research and Advanced Studies (ICREA), Barcelona, Spain; ^4^Department of History and Geography, University of Barcelona, Barcelona, Spain; ^5^Sant Joan de Déu Research Institute (IRSJD), Esplugues de Llobregat, Spain

**Keywords:** archaeoacoustics, spatial acoustics, experimental psychology, subjective evaluation, auralization, psychoarchaeoacoustics

## Abstract

How important is the influence of spatial acoustics on our mental processes related to sound perception and cognition? There is a large body of research in fields encompassing architecture, musicology, and psychology that analyzes human response, both subjective and objective, to different soundscapes. But what if we want to understand how acoustic environments influenced the human experience of sound in sacred ritual practices in premodern societies? Archaeoacoustics is the research field that investigates sound in the past. One of its branches delves into how sound was used in specific landscapes and at sites with rock art, and why past societies endowed a special significance to places with specific acoustical properties. Taking advantage of the advances made in sound recording and reproduction technologies, researchers are now exploring how ancient social and sacred ceremonies and practices related to the acoustic properties of their sound environment. Here, we advocate for the emergence of a new and innovative discipline, experimental psychoarchaeoacoustics. We also review underlying methodological approaches and discuss the limitations, challenges, and future directions for this new field.

## Introduction

How influential are the acoustics of surrounding space on the perception and interpretation of sound itself? Recent decades have seen a growing body of research that integrates architecture, music studies, psychology, and acoustical physics with the aim of unraveling how we relate to sound in space. Major progress in this area has come from the study of architectural acoustics of concert halls and the psychological effects on the audience, especially in terms of emotional responses (Beranek, 1962; Barron, 1971, 2009; Hawkes and Douglas, 1971; Schroeder et al., 1974; Lehmann and Wilkens, 1980; Farina, 2001; Farina et al., 2007; Lokki et al., 2010, 2011; Lokki, 2011; Pätynen and Lokki, 2011, 2016, 2018; Long, 2014). Progress has also stemmed from studying acoustics in cathedrals, churches, and synagogues (Lubman and Kiser, 2001; Abel et al., 2013; Markham and Azevedo, 2013; Álvarez-Morales et al., 2014; Pedrero et al., 2014; Postma and Katz, 2016a, b; Alonso et al., 2017; Pentcheva and Abel, 2017; Aletta and Kang, 2020), as well as acoustic environments, particularly focusing on noise pollution and human comfort (Cain et al., 2013; Davies, 2013; Davies et al., 2013; Hume and Ahtamad, 2013; Liu and Kang, 2015; Malecki and Piechowicz, 2016).

Beyond the study of the physics of sound and objective acoustic parameters, in psychology, this flourishing field mainly focuses on the participants’ subjective appraisal and their physiological and emotional responses to sound. The aesthetic experience of music is initiated and mediated by external and internal contexts, such as intentionality and background mood (Brattico et al., 2013; Menninghaus et al., 2019). The physical environment in which music is listened to or performed constitutes the most obvious external context. Some architectonically beautiful places with special acoustics seem to be optimal for determining the efficacy and intensity of an aesthetic musical experience and for inducing aesthetic awe (Koneĉni, 2008). Focusing on the subjective appraisal of sound has allowed psychologists to understand the influence of spatial acoustics in our reaction to sound and music, a reaction that is triggered pre-attentively (Frey et al., 2015, 2017). But what if our inquiry goes beyond trying to answer how soundscapes may have influenced the human experience of sound among premodern societies? An archaeological site itself may act as a musical instrument, generating sounds as a result of its own acoustical properties, or it may introduce a characteristic quality to other sounds made at or in the site (Till, 2014; Díaz-Andreu and Mattioli, 2019). The emergence of the field of archaeoacoustics over the past 20 years has led to a growing body of research to which psychology has scarcely contributed. One goal of this article is to enhance an interdisciplinary dialogue between psychology and archaeoacoustics. In this review, we argue in favor of a novel discipline, experimental psychoarchaeoacoustics, and offer some basic tenets and methodological approaches for it, while, at the same time, discuss its limitations and potential challenges.

## Archaeoacoustics

The goal of *archaeoacoustics* is to investigate how sounds influenced the human experience in the past (Scarre and Lawson, 2006). It is a field in which there has been a sharp increase in interest in recent years. How was sound used in specific landscapes or sites? Why did ancient societies endow a special significance on selected places with specific acoustical properties? What kind of sounds did they create with instruments [either natural, such as sonorous stones, or artificial, such as bone flutes and drums (Hultman, 2014)] or even their own voices? These are typical questions posed by scholars interested in this multidisciplinary territory, which brings together acoustics and archaeology, as well as architecture, engineering, and neuroscience. Here, we will focus on research undertaken into the relationship between acoustics and rock art landscapes, i.e., landscapes where paintings and/or engravings were made in the distant past. The aim of this research is to discern whether specific landscapes and sites with rock art were selected for their special acoustic properties and to ascertain what effects these could have elicited in listeners. Rock art sites have been seen as places with no immediate economic function, i.e., but rather as places with an important sacred component. Anthropologists and ethnomusicologists have suggested that there can be no ritual without music (Tuzin, 1984; Bloch, 1989; Nettl, 2000), and that may well be why rock art creators favored places with particular acoustics. Researchers have examined the connection between acoustics and rock art in relation to the location of rock art in naturally sonorous landscapes, as well as in landscapes where artificially produced sounds can induce relevant acoustic responses. This type of inquiry differs from others in archaeoacoustics aimed at exploring the kinds of sounds ancient communities were able to produce with musical instruments and their own voices (Sánchez, 2007; Kleinitz, 2008; Kollveit, 2008). It also differs from the studies that examine the intentional creation of special acoustics in the construction of buildings such as pyramids, theaters, or cathedrals (Lubman, 1998; Declercq et al., 2004; Watson, 2008; Abel et al., 2013; Pentcheva and Abel, 2017; Kolar, 2018). The emphasis in the study of rock art soundscapes is on acoustics in natural landscape settings.

Interest in the sound and acoustics of places used by communities in pre-states societies (from hunter-gatherers to pre-industrial producing economies) grew in archaeology in the late 1980s. It progressed from early studies of lithophones in Paleolithic caves with art (Reznikoff, 1987; Reznikoff and Dauvois, 1988; Waller, 1993a) to those in landscapes. This shift in focus began in the United States (Steinbring, 1992, 1993; Conway, 1993; Hedges, 1993; Waller, 1993b) and later spread to other places in the world, including Finland (Reznikoff, 1995; Lahelma, 2010; Rainio et al., 2014), Siberia (Jacobson and Kubarev, 1994), South Africa (Ouzman, 2001), Scandinavia (Goldhahn, 2002), and the Mediterranean (Díaz-Andreu and García Benito, 2012; Mattioli et al., 2017), with subsequent new work being undertaken in the US (Waller et al., 1999; Waller, 2000) and with cave art (Fazenda et al., 2017). The studies focused on acoustic effects such as echoes and the audibility of distant sounds. The research on echoes in Horseshoe Canyon demonstrated that its rock paintings had been located at points where echoes were most intense. The direct relationship between echoes with a high dB strength and the placement of rock art led to the finding of a new rock art site where the model had predicted it would be (Waller, 2006). Spirituality and human beliefs were also related to the natural landscape. In Quebec, the First Nations believed that rock art sites located close to water were occupied by *memegwashio*, small, hairy, mythical, human-like creatures (Arsenault, 2004). The fieldwork undertaken into acoustics revealed that there was indeed a direct relationship between those areas and distinct acoustic properties, especially echoes (Waller and Arsenault, 2008). In northern Finland, there are prehistoric rock paintings (5,200 to 1,000 BC) and an ancient Sámi offering site (*circa* 1,100 to present) near the canyon lakes of Julma-Ölkky, Somerjärvi, and Rotkojärvi. An archaeoacoustics research project explored the role of sound in the development and use of these sites. Using methods such as multichannel impulse response (IR) recording, angle-of-arrival estimation of early reflections, spectrum analysis, digital image processing, and 3D laser scanning, the researchers concluded that the cliffs with rock paintings are efficient sound reflectors (Rainio et al., 2018). The sound appears to emanate directly from the painted figures. The audibility of distant sounds has also been suggested as one of the reasons for the location of rock art in particular areas (Mattioli and Díaz-Andreu, 2017). Rock art researchers have been looking at the reasons behind the relationship between sound and ritual. Some propose the existence of universals to explain the importance of acoustics as a way of facilitating the connection between humans and spiritual beings and/or ancestors (Waller, 2002). In other cases, the use of ethnohistorical sources in combination with other techniques has also proved to be useful (Díaz-Andreu et al., 2020). In any case, the effects of acoustics on several informational aspects of sound patterns, such as pitch, timbre, rhythm, and melody, both in speech and music, may have played a key role (Bidelman and Krishnan, 2010; Bidelman, 2017; Eurich et al., 2019). Neural representation of pitch based on timing information is severely degraded in the presence of reverberation (Sayles and Winter, 2008). Sentence intelligibility is also adversely affected by reverberation and nonstationary background noise (George et al., 2008). These types of findings could partially explain how the loss of speech intelligibility through reverberation and echo may have been key to the ensoulment of rock art sites: by depriving the speaker’s speech of its natural intelligibility, the heard sound may have been perceived as having come from a different “speaker” (i.e., the voice of the site), thus endowing it with its sacred significance.

To test the acoustics at rock art sites, researchers have used different sound stimuli covering a wide range of sources, including the human voice (Reznikoff, 1987; Reznikoff and Dauvois, 1988) and single loud percussion noises *via* a spring-loaded device (Waller 2002). Other articles describe the use of a combination of sounds: human voice (male, female, or both simultaneously) uttering the vowel “a”; handclapping for around 5 s; and whistles of different musical tones (between the ranges of C_5_/C#_5_ and G_5_/G#_5_, due to the effect created by the air speed during the fieldwork), played together to obtain a fifth interval and alone (G_5_/G#_5_), producing intermittent sounds (Díaz-Andreu and García Benito, 2012). Nevertheless, the use of a sine sweep (a sine function that gradually changes frequency over time) covering all human audible frequencies, allows a more reliable way to dynamically characterize its IR. IR is the acoustic fingerprint of a space, and provides a measurement of sound propagation between an emission point and a receiver device that is usually located within the same environment (Farina et al., 2007; Kuttruff, 2009). This is an example of the use of more precise methodologies borrowed from acoustical physics, which has allowed more accurate results to be obtained. In direct relation to sound stimuli delivery, different techniques, such as binaural recording (Rainio et al., 2014) or Ambisonics recording (Mattioli et al., 2017), have been used in the study of rock art soundscapes to analyze the propagation of speech and music in these sites. Although binaural techniques cover in many cases the experimental needs, the Ambisonics technique produces more precise measurements of angular readings of the direction of arrival of echoes and reverberation than the binaural technique (Mattioli and Díaz-Andreu, 2017; Díaz-Andreu et al., 2020), and the use of sine sweeps provides, compared to other forms of stimulation, a more comprehensive characterization of the acoustic signature of a sonic space.

In addition to rock art studies, the work on archaeological psychoacoustics undertaken at the renowned pre-Inca site of Chavín de Huántar in Peru is also relevant to our approach. It is pertinent here to clarify the concept of psychoacoustics as used by those researchers. Psychoacoustics is the science of the hearing system as a receiver of acoustical information (Fastl and Zwicker, 2007). In this experiment, however, the authors expanded this view to include the participants’ subjective perception of sound. At the Chavín de Huántar, the sonic environment of the site’s ancient mortared-stone complex was characterized using a swept exponential sinusoidal test signal from an emitter positioned at Chavín-period human head height. Recordings were made at two sets of receivers to retrieve the reverberation time (RT), echo density, interaural cross correlation coefficients, and lateral energy fraction (Abel et al., 2008). These data were subsequently used to create a computational acoustic model of the interior architecture of the ceremonial site (Collecchia et al., 2012). An on-site psychoacoustics experiment was also carried out in which 45 volunteer participants were asked to locate sound sources within the Chavín interior galleries. Although the precise methodological details and outcomes of that experiment have not been published, the author reported that “participant responses to systematic testing of sound source/receiver ‘perceiver’ location combinations demonstrated broad consensus regarding the perceptual effects of gallery acoustics on sound transmission, providing verifiable ‘mappings’ of sonic communication dynamics within those spaces” (Kolar, 2017). The same authors later developed an archaeoacoustics method that compared a sequence of human-performed sound sources, along with a standard electronic acoustical test signal, across survey points at the Inca administrative complex of Huánuco Pampa. The authors combined ecologically valid acoustical measurements with subjective researcher-observer data to chart sound transmission and reception of different classes of sounds, enabling the identification of environmental contingencies, and the estimation of site acoustical features (Kolar et al., 2018).

## Experimental Psychoarchaeoacoustics

Here, we want to advocate for the emergence of novel discipline of inquiry: experimental psychoarchaeoacoustics. It will be experimental because it will attempt to be reproduced in the laboratory, in a controlled and replicable manner, the acoustic conditions encountered at archaeological sites. It is related to the field of psychology because we are interested in the human experience with sounds and their modulation by different environmental acoustics, including issues such as how sounds are perceived and the type of emotional feelings, and even the mental states they may induce. These experiments in soundscape psychology will be connected to archaeology, given that we are interested in the motivations of past individuals in acoustics and sound. In our case study, our focus will be on what led people to paint or engrave rock art at sonorous sites in the distant past. We wish to inquire into perception and emotion in the past related to sound. The use of laboratory acoustic experiments includes new variables and potential confounds, but allows detailed control of the intervening processes. While experiments in the laboratory will lack fidelity and ecological validity, they will offer flexibility and control of the independent variables (Rossing, 2007).

The use of multichannel audio for the reproduction or simulation of multidimensional sound fields is becoming common in research and other fields of human endeavor, such as artistic performances or commercial installations (Guastavino and Katz, 2004). This is made possible by improvements in technology over the years, together with the appearance of new recording techniques able to reproduce sound fields. Seminal research has also been newly carried out with sound fields generated by convolving anechoic music with binaural IRs from actual concert halls. In an already more-than-two-decades-old research, participants were asked to listen to the resulting sound fields over a pair of loudspeakers over eight separate tests. They were instructed to make judgments based on the perceptual differences between pairs of sound fields (Soulodre and Bradley, 1995). Since then, the precise reproduction of the acoustics of a concert hall, sound environment or building in a laboratory, known as *auralization*, has become a very common approach in lab-based acoustics research (Vorländer, 2008). This is the approach taken in our proposal.

Auralization is a very powerful technique for evaluating sound and acoustics perception in the laboratory (Lokki, 2002; Pätynen, 2011; Pätynen and Lokki, 2011; Vorländer, 2014; Postma and Katz, 2015). It allows audible sound files from simulated or measured data to be created and reproduced using loudspeakers or headphones (Vorländer, 2008). However, to recreate how a real space sounds, it is necessary to retrieve its acoustic signature. Thus, to obtain parameters such as RT or clarity, it is common to use the so-called IR (Kuttruff, 2009). As we described above, IR measures sound propagation between a sound emission point and a receiver device. Rock art landscapes, however, are not concert halls; in them sound propagation is affected by complex reflections, diffractions, and absorptions mainly caused by natural hard surfaces of the geology and the vegetation in the area. All those aspects need to be considered when placing the emitter and the receiver in the landscape. Also in relation to experimental design, to study the effects of the soundscape on the psychological response of participants, tests should be not only be undertaken at sites of interest, but also at control sites with similar morphological and geographical features, but no rock art. Auralization needs the acoustic signature of a space and a dry sound stimulus as inputs. A mathematical approach known as convolution is used to convey the dry signal through the acoustic signature and results in a simulation of how the sound stimulus would be heard in the landscape being analyzed. These sounds then have to be reproduced using one of many audio systems in a prepared listening room (Pätynen and Lokki, 2016). Convolution also allows the production of artificial reverberation imprinting of the IR of a space onto an input audio signal (Pentcheva and Abel, 2017).

There are several sound reproduction systems available for the implementation of psychoarchaeoacoustic experiments. A loudspeaker-based auralization reproduces the sound field at the listener’s location in the center of the loudspeaker array (Guastavino and Katz, 2004; Favrot, 2010). A headphones system needs to consider specific individual listener information such as individual Head-Related Transfer Function (HRTF; Favrot and Buchholz, 2010). Ambisonics reproduction systems are three-dimensional sound reproduction systems that simulate the sound field at any given point in the room (Howard and Angus, 2006). Binaural processing aims to generate the same spatial sensation only by using a couple of headphones or two loudspeakers with cross-talk cancellation to disable any spatial cues that may be present and to restrict the resulting sound image to the confines of the loudspeaker placement (Lorho, 2010).

Several options have been proposed for procedures to rate subjective sound perception (Schroeder et al., 1974; Soulodre and Bradley, 1995; Hidaka and Beranek, 2000; Västfjäll et al., 2002; Bishop and Rohrmann, 2003; Pätynen, 2011; Vigeant et al., 2011; Cain et al., 2013). Listening tests are experimental procedures in which a group of participants is asked to respond to certain questions regarding sound stimuli they have to listen to. Listening tests are methods for the subjective evaluation of perception in acoustics and they are typically used to evaluate how humans perceive spaces and how this perception can affect their actions (Prida et al., 2019). Unlike objective parametrization, listening tests have proved their effectiveness as methods for the subjective assessment of perception in several types of rooms and different acoustic fields (Cox et al., 1993; Soulodre and Bradley, 1995; Bradley et al., 1999; Guastavino et al., 2005; Witew et al., 2005; Kolarik et al., 2013; Postma and Katz, 2016b). There may even be discernable neural signatures for different kinds of acoustic spaces (Teng et al., 2017).

The goal of rating subjective sound perception is to ascertain what users perceive and how they evaluate sounds (Susini et al., 2013). Three different approaches can be undertaken to evaluate sound-induced emotions, depending on whether the interest is in emotion-related subjective, behavioral, or physiological levels (Levenson, 1994). For subjective evaluation of sound or sound fields, participants rate their consciously felt emotional experience through self-reports that can be classified into verbal and visual tests (Tajadura-Jiménez, 2008). Verbal self-reports are created by means of descriptions of feelings/attitudes with answers that can be given using different types of rating scales, which could include open-ended questions and interviews. Examples of rating scales for basic emotions are the *Mood Adjective Checklist* or MACL (Nowlis, 1965), the *Multiple Affect Adjective Check List* or MAACL (Zuckerman et al., 1965), the *Profile of Mood States* or POMS (McNair et al., 1971), and the *Differential Emotions Scale* or DES (Izard et al., 1993). In relation to characterizing effect, example solutions are the *Positive Affect/Negative Affect Measure* or PANAS (Watson et al., 1988), the *Activation-Deactivation Adjective Checklist* or AD-ACL (Thayer, 1967), and the *Current Mood Questionnaire* or CMQ (Barrett and Russell, 1998). Visual self-reports are easy to interpret and relatively unambiguous across cultures. Examples of tests are the *Affect Grid* (Russell et al., 1989), the *Self-Assessment Manikin* (Bradley and Lang, 1994), and the *Product Emotion Measurement Instrument* (Desmet, 2002). Physiological measurements involve recording changes in biological systems that are caused by emotional processes (Tajadura-Jiménez, 2008). The most common non-invasive techniques are the measurement of electrodermal activity (EDA), heart rate (HR), facial electromyography (EMG), and electroencephalographic activity (EEG) (Cacioppo et al., 2016).

## Challenges For a New Discipline

While the endeavor set forth in this review is appealing and well grounded, it is not of course free of challenges. These are mainly related to methodological issues, although there are also epistemological limitations in the disciplines involved. An example of the latter is the initial hypothesis that ancient rock art sound environments are endowed with special acoustics and that these acoustic properties enhanced sacred practices for which sound was important. A specific mystical experience does not only rely on a cultural dimension, although ultimately the results related to biological universals could lead to the understanding of common structures prone to this mystical experience (Krueger and Grafman, 2012; Maselko, 2012; Cristofori et al., 2016). There is also a need for an interdisciplinary discussion regarding the cultural dimension of acoustic parameters extracted from an archaeological site. Echo can be considered a physical phenomenon that encapsulates reflection and diffraction and is posited on a material level, but it can also act on a symbolic level as a sounding disturbance in traditional subject-object relations (Goh, 2017).

Obtaining an IR in a selected sound environment involves considering control conditions for making comparisons, as well as taking into account the location of the sound emitter and receiver in it. Unlike cathedrals or churches, in natural sound environments there are no physical clues as to where the songs where performed and listened to, or from where mythical stories were narrated and/or speeches delivered. This is a handicap that can be partially overcome by placing the emitter and receivers in several different positions. Another limitation emerges when the historical heritage has disappeared or degraded over time. An archaeoacoustics study of the Ħal Saflieni Hypogeum in Malta found methodological limitations due to changes made to the site over time that affected the acoustics (Till, 2017). Given that much of the archaeological context was missing, any interpretative conclusions drawn were limited. In these cases, researchers can try to recover the original acoustics of the site through documentation of the place, computer modeling tools, and simulation approaches (Rua and Alvito, 2011; Suárez et al., 2016). In general, modeling tools in archaeoacoustics can be used to model soundscapes and to explore how people heard their surroundings without the natural limitations of real places. One common tool in archaeology is GIS technology. Modeling the spread of sound in a GIS environment places emphasis on the spatial location and the extent of the soundshed (the geographical area that is audible from a location) rather than on a detailed acoustical reconstruction (Witt and Primeau, 2018). An example of this use can be found in the investigation of the Chaco Canyon acoustics, where the output of a GIS soundshed analysis tool described the propagation patterns of sound throughout the landscape and the increase over ambient sound pressure level (Primeau and Witt, 2018). However, all the models obtained when using these tools involve many assumptions by the software operators in their variables and datasets. This limitation adds an important degree of uncertainty to the output. Prediction of RTs with an accuracy greater than the just noticeable difference requires the input of data of a quality that is not available from reverberation space measurements (Vorländer, 2013).

Selection of the sound stimuli is also a relevant aspect of the experimental design. This is not a major problem in the case of subjective analyses of the responses of concert hall acoustics test participants, as in those experiments the music heard is that commonly played in those venues (Pätynen, 2011; Lokki, 2014). In contrast, studying premodern music practices involves deciding between three different sets of stimuli: contemporary music, music excerpts attributed by ethnomusicologic research to premodern societies, or other kinds of non-musical sounds (e.g., birdsong; Fitch, 2006; Benichov et al., 2016). Choosing musical stimuli (from the present or the past) often involves neglecting any potential effect of culture on musical listening but makes it more natural for participants. In particular areas, the solution could be to explore the acoustic properties of present-day sacred sites with rock art that are currently being used by tribal communities. Using a non-musical stimuli overcomes any culturally driven emotion but lacks naturalness. There is a potential need to identify ecologically valid sound sources and preference universals (Mühlenbeck et al., 2017) when investigating participants’ subjective parameters. In cases where an objective characterization is the only requirement (i.e., the number and type of echoes), simple stimuli such as clicks could be used.

In addition to the selection of stimuli, a second research challenge to overcome is to undertake the auralization in an acoustically prepared space. The listening room has to allow for a wide array of simulated RT that could eventually range from tenths of a second to tens of seconds (Kuttruff, 2009; Favrot, 2010). To this end, the actual RT of the space cannot be high to allow for simulating spaces with a lower value. However, nor can it be close to zero, as in a hemi-anechoic or completely anechoic chamber, as the participants usually experience discomfort due to not hearing the reflections that would usually be produced in the room (Wenzel et al., 2017). Regarding the hearing of reproduced spatial sounds, there is a need to distinguish the attributes of the source environment from those that are governed by the recording technique, and from those influenced by the reproduction system and the listening room (Rumsey, 1998).

A final challenge facing experimentation in psychoarchaeoacoustics is related to the participants’ body posture, movements, and place illusion. A listening experiment of this kind will usually be carried out with participants sitting or standing quietly at a given place in the room. A surround loudspeaker system involves not moving the head from the optimal listening position at the center of the setup. Using a headphone system means that the horizontal and vertical axis will move with head movements if extra sensors are not placed to detect and correct head rotation. These constraints make experiments, such as navigating the space, performing ritual movements, or dancing in more naturalistic setups, very challenging, if not impossible. Moreover, the acoustic simulation of a soundscape may be poor if not accompanied by visual cues (Kleiner et al., 2002). This can be remedied by using a virtual reality system to enhance the multisensory experience by creating the illusion of a setting of any size and populated by any number of individuals, if needed (Slater, 2009). Also, simulated wind created by controlled fans would allow researchers to investigate its role in the soundscape.

As can be seen, experimental psychoarchaeoacoustics is an emerging field that faces many challenges, yet we deem it to be a feasible new field. There are encouraging experimental results in the study of the influence of acoustics in emotional impact in concert halls (Lawless and Vigeant, 2015; Pätynen and Lokki, 2016). As we have discussed along the review, the same evidence based principles are applicable in the study of archaeological sites when measuring a site acoustics, when developing their auralizations in the laboratory and when measuring participants’ responses. The most important methodological challenges have been discussed above. Other challenges are epistemological and these are ultimately more difficult to overcome from a perceptual and psychological point of view. However, they are at the heart of archaeological endeavor, given that we do not have access to actual data regarding past communities’ practices, rituals, and culture. Interrogating contemporary people on how they feel about sounds played with the acoustics of particular archaeological sites can reveal the influence of different acoustics on modern people’s perceptions and emotions. Furthermore, rock art was produced by early hominids similar to us and, therefore, possessing similar core mechanisms (neurological, psychological, etc.) leading to similar perceptual and emotional experiences. A key assumption underlying the proposed field of experimental psychoarchaeoacoustics is that the effects of soundscape acoustics on human experience are universal in ancient and modern times and listeners. These experimental data gathered from modern listeners in auralized sacred sites can lead to insights as to how “sacred sonorous” spaces are perceived by participants and answer whether there are acoustic properties common to all rock art soundscapes. There is no evidence that modern listeners in a virtual “ancient sacred soundscape” will really feel spiritually moved, but with the obtained data we could infer the potential effects of sound and acoustics in human psychology.

## Author Contributions

JV was the primary author of this study, he wrote the mini-review and received feedback from each of the other supervisors. MD-A and CE were supervisors as part of this research topic. Each of the supervisors provided insight, expertise, and feedback on the paper. They provided several edits and proofread throughout the refinement of the article. All authors contributed to the article and approved the submitted version.

### Conflict of Interest

The authors declare that the research was conducted in the absence of any commercial or financial relationships that could be construed as a potential conflict of interest.
